# A Tale of Switched Functions: From Cyclooxygenase Inhibition to M-Channel Modulation in New Diphenylamine Derivatives

**DOI:** 10.1371/journal.pone.0001332

**Published:** 2007-12-26

**Authors:** Asher Peretz, Nurit Degani-Katzav, Maya Talmon, Eyal Danieli, Anna Gopin, Eti Malka, Rachel Nachman, Amiram Raz, Doron Shabat, Bernard Attali

**Affiliations:** 1 Department of Physiology and Pharmacology, Sackler Faculty of Medicine, Tel-Aviv University, Tel Aviv, Israel; 2 Department of Biochemistry, Georges Wise Faculty of Life Sciences, Tel-Aviv University, Tel Aviv, Israel; 3 School of Chemistry, Faculty of Exact Sciences, Tel-Aviv University, Tel Aviv, Israel; The Scripps Research Institute, United States of America

## Abstract

Cyclooxygenase (COX) enzymes are molecular targets of nonsteroidal anti-inflammatory drugs (NSAIDs), the most used medication worldwide. However, the COX enzymes are not the sole molecular targets of NSAIDs. Recently, we showed that two NSAIDs, diclofenac and meclofenamate, also act as openers of Kv7.2/3 K^+^ channels underlying the neuronal M-current. Here we designed new derivatives of diphenylamine carboxylate to dissociate the M-channel opener property from COX inhibition. The carboxylate moiety was derivatized into amides or esters and linked to various alkyl and ether chains. Powerful M-channel openers were generated, provided that the diphenylamine moiety and a terminal hydroxyl group are preserved. In transfected CHO cells, they activated recombinant Kv7.2/3 K^+^ channels, causing a hyperpolarizing shift of current activation as measured by whole-cell patch-clamp recording. In sensory dorsal root ganglion and hippocampal neurons, the openers hyperpolarized the membrane potential and robustly depressed evoked spike discharges. They also decreased hippocampal glutamate and GABA release by reducing the frequency of spontaneous excitatory and inhibitory post-synaptic currents. *In vivo*, the openers exhibited anti-convulsant activity, as measured in mice by the maximal electroshock seizure model. Conversion of the carboxylate function into amide abolished COX inhibition but preserved M-channel modulation. Remarkably, the very same template let us generating potent M-channel blockers. Our results reveal a new and crucial determinant of NSAID-mediated COX inhibition. They also provide a structural framework for designing novel M-channel modulators, including openers and blockers.

## Introduction

Cyclooxygenase (COX) enzymes catalyze the conversion of arachidonic acid into prostaglandin H2 (PGH2), the early step in the biosynthesis of prostanoids [1]. The COX enzymes are functional homodimers, where each subunit contains both a cyclooxygenase and a peroxidase active site. Three COX isozymes have been characterized so far, COX-1, COX-2, and COX-3. While COX-1 is a ubiquitous constitutive form of the enzyme that is involved in the regulation of various physiological processes such as platelet aggregation or homeostasis of the gastrointestinal tract and kidneys, the COX-2 isozyme expression is mainly observed during inflammatory processes [1], [2]. COX-2 is constitutively expressed in some tissues, but can be induced by different stimuli such as growth factors or interleukin-1. All NSAIDs inhibit the COX isozymes to different extents, which accounts for their anti-inflammatory and analgesic properties as well as their gastrointestinal side effects, the latter being more related to COX-1 inhibition [3]. Hence, the development of COX-2-selective inhibitors as nonulcerogenic, anti-inflammatory, and analgesic agents was greatly stimulated. However, the recent withdrawal of diarylheterocyclic selective COX-2 inhibitors due to adverse cardiovascular side effects toned down the initial enthusiasm surrounding the launch of selective anti-COX-2 drugs [4]–[6].

The COX enzymes are not the sole the molecular targets of NSAIDs. Recently, we showed that two chemically-related NSAIDs, diclofenac and meclofenamic acid also act as openers of Kv7.2/3 (KCNQ2/3) potassium channels [7], shown to underlie the neuronal M-currents. The M-channels generate a subthreshold, noninactivating voltage-gated K^+^ current that plays an important role in controlling neuronal excitability [8]. M-channels are thought to mediate early spike frequency adaptation by producing a medium after-hyperpolarization (mAHP) [8]–[11]. M-currents also activate during the spike afterdepolarization (ADP) limiting its amplitude and duration, thus preventing its escalation to a burst [12]. Reflecting their physiological importance, mutations of the Kv7.2 and Kv7.3 genes have been identified as causes of myokymia and of benign familial neonatal convulsions, a neonatal form of epilepsy [10]. Pharmacological targeting of M-channels is of great clinical importance. While openers demonstrate a therapeutic potential for the treatment of neuronal hyperexcitability like migraine, epilepsy and neuropathic pain, blockers are potentially useful for the treatment of memory deficits and Alzheimer's disease [13], [14]. Here, we generated a series of novel derivatives of diphenylamine carboxylate to discriminate the M-channel modulatory activity from the COX inhibition. For this purpose, we derivatized the carboxylate functionality into amides or esters which were attached to different alkyl and ether chains. We obtained potent M-channel openers with or without COX inhibition. Conversion of the carboxylate moiety into amide successfully removes the COX inhibition but preserves the M-channel modulation. Our data disclose an important and novel determinant of COX inhibition by the diclofenac and N-phenylanthranilic acid NSAID subfamilies. A structural framework is now available for the design of novel and selective M-channel modulators and for tailoring independently potent new NSAIDs.

## Results

### Carboxylate derivatization of diclofenac and meclofenamic acid

Knowing the non-selective inhibition of COX-1 and COX-2 enzymes displayed by diclofenac and meclofenamic acid [15] as well as their recently described M-channel opener properties [7], our purpose was to design potentially powerful M-channel openers, devoid of substantial anti-COX activity. The carboxylate group of diclofenac and meclofenamic acid was derivatized into amides or esters and linked to different alkyl or ether chains of various lengths (Table 1 and Table S1). In addition, the aromatic rings of the diphenylamine moiety were substituted with different electron donating or withdrawing groups. In a first screen, two parameters were checked: (a) the non-selective inhibition of COX-1/COX-2 enzyme activities, measured by the production of PGE2 in cultured mouse colon adenocarcinoma (CT26) and mouse Lewis lung carcinoma cells (D122) cells; (b) the M-channel activity, measured by the heterologous coexpression of Kv7.2 and Kv7.3 subunits in Chinese hamster ovary (CHO) cells.

**Table 1:** SAR studies of derivatives of diclofenac and meclofenamic acid.

Compound	COX inhibition (IC_50_, µM)		M-channels (Kv7.2/Kv7.3)	ΔV_50_ (mV)
	CT26	D122		
Diclofenac	0.003	0.1	opener (EC_50_ = 2.6 µM)	−14.5
Meclofenamic acid	nd	nd	opener (EC_50_ = 25 µM)	−22.7
1	>50	>50	opener	nd
2	40	30	opener (EC_50_ = 7 µM)	−12.6
3	nd	nd	opener	nd
4	10	15	opener	nd
5	nd	nd	opener	nd
6	>50	>50	opener (EC_50_ = 14 µM)	−31.3
7	>50	>50	opener (EC_50_ = 17 µM)	−12.2
8	0.015	0.003	opener (EC_50_ = 18 µM)	−18.7
9	0.010	0.001	opener	−4.1
10	nd	nd	inactive	
11	nd	nd	blocker	
12	nd	nd	inactive	
13	0.001	0.010	blocker	
14	nd	nd	blocker	
15	0.048	0.229	opener (EC_50_ = 22 µM)	−31.0
16	20	5	opener (EC_50_ = 3 µM)	−8.4
17	20	50	opener (EC_50_ = 9 µM)	−5.5
18	0.5	30	opener (EC_50_ = 6 µM)	−3.7
19	nd	nd	inactive	
20	nd	nd	inactive	

COX inhibition was measured by the production of PGE2 in cultured mouse colon adenocarcinoma (CT26) and mouse Lewis lung carcinoma cells (D122) cells. IC_50_ values in µM represent the average values from two experiments, each performed in triplicate. The impact of the compounds on M-channel activity was measured by the heterologous coexpression of Kv7.2 and Kv7.3 subunits in CHO cells. EC_50_ values in µM represent the average values of opener potency as determined by the concentration-dependent left-shift in the half-activation potential ΔV_50_ (mV) and fitted by a sigmoidal function (n = 3–5).

**Figure 1:**
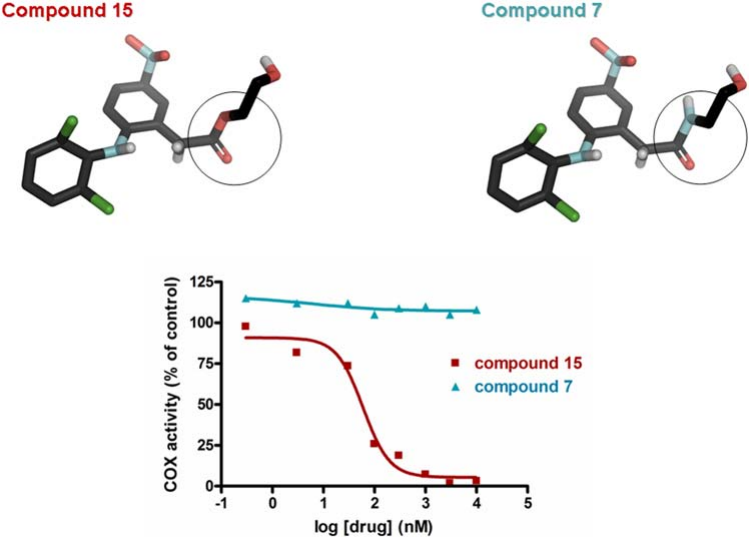
Inhibition of COX activity by compounds 7 and 15. The inhibition of COX enzyme activity was measured by the production of PGE2 in cultured mouse colon adenocarcinoma cells (CT26). While the COX activity is inhibited by the ester compound 15 (IC50 = 69 nM), it is completely unaffected by the amide compound 7. This typical experiment gave similar results in two additional trials. Shown are the chemical structures of compounds 7 and 15.

Considering the COX activity, it is striking to note that all the amide derivatives of diclofenac (compounds 1, 2, 4, 6 and 7) and meclofenamic acid (compounds 16–18) produced very weak or no inhibition of the COX enzymes in CT26 and D122 cells (IC_50_ >50 µM; Table 1 and Table S1). This virtual lack of inhibition of the COX activity was displayed by amides derivatives bearing ether as well as alkyl chains (e.g., compounds 2 and 7). While the issue of the COX selectivity was not directly addressed in this study, we tentatively assume that the lack of COX inhibition displayed by the various amide compounds involves both COX-1 and COX-2, since the D122 cell line is endowed with both COX-1 and COX-2 activities and the CT26 line expresses mainly though not exclusively COX-2 [16], [17]. These features contrast with the well known anti-COX-1/COX-2 properties of the parental templates [15]. In contrast, all ester compounds tested, produced a potent inhibition of the COX enzymes (IC_50_s∼1 to 230 nM) (Table 1 and Table S1). The prominent difference in COX inhibition between amide and ester derivatives is well illustrated with compounds 2 and 8 or with compounds 7 and 15 (Table 1, Table S1 and Figure 1). Comparison of compounds 8 and 13 suggests that the terminal hydroxyl functionality of the ester derivatives is not important for the anti-COX activity. Compound 13 is even more powerful than the parent diclofenac template. Similarly, a potent and non-selective inhibition of purified COX-1 and COX-2 enzymes was obtained with compounds 8 and 13 and diclofenac. Thus, at 1 µM concentration compounds 8, 13 and diclofenac respectively produced 58±1%, 59±1% and 64±2% inhibition of the purified ovine COX-1 enzyme (n = 3) and 85±1%, 83±1% and 72±2% inhibition of the human recombinant COX-2 enzyme (n = 3).

**Figure 2:**
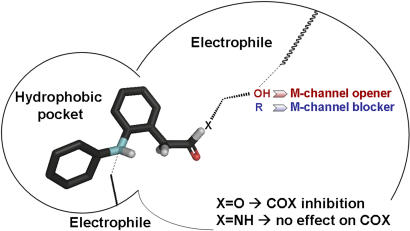
Pharmacophoric features of M-channels deduced from SAR studies of diphenylamine derivatives.

Considering the M-channel activity, several important features could be deduced from the structure-activity relation (SAR) study (Table 1, Table S1 and Figure 2). In contrast to the dramatic impact of the carboxylate conversion into amide on COX inhibition, the M-channel openers can clearly accommodate with carboxylate, ester and amide functionalities. Table 1 and Table S1 show that openers of the Kv7.2/3 channels are found in compounds bearing a carboxylate (e.g., compound 3, diclofenac, or meclofenamate), an ester group (e.g., compounds 8, 9 and 15) as well as an amide function (e.g., compounds 2, 6, 7 and 16). The opener activity is also displayed by derivatives bearing either ether or alkyl chains (e.g., compounds 4–7 and 2 or 8 and 9). As the main opener effect of the various compounds is to cause a hyperpolarizing shift of the activation curve of Kv7.2/3 channels, the extent of the left-shift (ΔV_50_) is a faithful measure of the opener potency (Table 1 and Table S1). Hence, we found that substituting the aromatic rings with NO_2_ groups significantly increased the left-shift in activation. In the ester series, compound 9 is a much weaker opener (ΔV_50_ = −4.1 mV) than compound 15 which bears one NO_2_ (ΔV_50_ = −31.0 mV). Similarly, in the amide series, compound 6 possessing three NO_2_ is a stronger opener (ΔV_50_ = −31.3 mV) than compound 7 which bears only one NO_2_ (ΔV_50_ = −12.2 mV).

**Figure 3:**
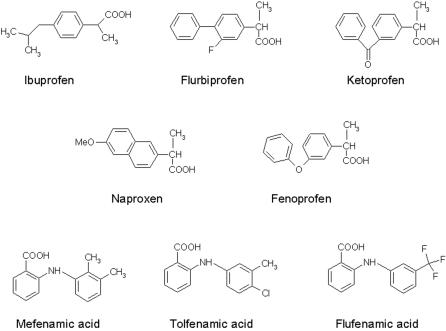
Chemical structures of NSAIDs lacking the diphenylamine moiety but still bearing a carboxylate function like ibuprofen, flurbiprofen, ketoprofen, fenoprofen and naproxen. Chemical structures of N-phenylanthranilic acid drugs containing the diphenylamine moiety like mefenamate, tolfenamate and flufenamate.

**Figure 4:**
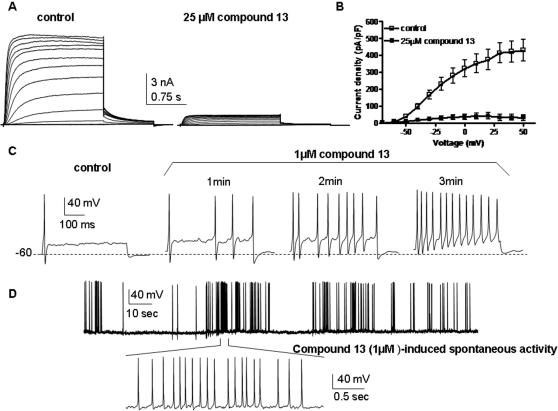
Compound 13 inhibits Kv7.2/3 currents and enhances firing of peripheral DRG neurons. (A) Representative traces recorded from the same CHO cell before (left panel) and after (right panel) external application of compound 13 (25 µM). The membrane potential was stepped from −90 mV (holding potential) to +50 mV for 1.5 s pulse duration in 10 mV increments, followed by a repolarizing step to −60 mV. (B) Current density-voltage relations in the absence (empty squares) and presence of compound 13 (25 µM) (solid squares) (n = 6). (C) Representative rat DRG spiking discharge, evoked by a squared depolarizing current pulse (10 pA for 400 msec) before (control), during exposure to 1 µM compound 13 for 1, 2 and 3 min. (D) Representative trace of spontaneously spiking DRG neuron previously exposed (5 min) to 1 µM compound 13.

The diphenylamine moiety itself appears to be important for activating the M-channels. We found that NSAID compounds lacking this group but still holding a carboxylate function like ibuprofen, flurbiprofen, ketoprofen, fenoprofen or naproxen, do not activate Kv7.2/3 channels, while those bearing both functionalities (diphenylamine and carboxylate) such as N-phenylanthranilic acid drugs (mefenamate, tolfenamate, or flufenamate) are all M-channel openers (Figure 3). Noteworthy, our data indicate that a terminal hydroxyl group in the ether or alkyl chain is absolutely required to obtain an active M-channel opener. For example, the compounds 10, 12, 19 and 20 whose terminal hydroxyl function has been replaced by methyl or isobutyl groups are totally inactive vis-à-vis Kv7.2/3 channels (Table 1, Table S1 and Figure 2). Remarkably, in the diclofenac series we obtained potent M-channel blockers with compounds 11, 13 and 14 where the terminal hydroxyl has been replaced by isobutyl, ethylamine and 2,3-epoxypropyl groups, respectively (Table 1 and Table S1). When tested on recombinant Kv7.2/3 channels that were heterologously expressed in CHO cells, compound 13 potently and dose-dependently produced a blockade of the K^+^ currents (IC_50_ = 11 µM). At 25 µM compound 13 produced more than 90 % inhibition of the Kv7.2/3 current at a wide range of membrane potentials (from −60 to +50 mV; Figure 4A and B). Recent work suggested that M-currents play a key role in controlling the excitability of sensory dorsal root ganglion (DRG) neurons and may therefore represent a therapeutic target for the treatment of pain [18]. Hence, measuring the effects of novel M-channel modulators on DRG neuronal excitability is of important value, considering their potential impact on nociceptive signaling pathways. We examined in the current-clamp configuration the effects of compound 13 on spike activity of cultured rat DRG neurons (Figure 4C). A single spike discharge pattern was evoked by injecting a minimal depolarizing current pulse (∼10 pA, 400 ms). Within 2 min, external application of 1 µM compound 13 depolarized the DRG membrane potential (ΔV = +7±1 mV) and potently increased the number of evoked spikes (∼10–20 pA for 400 ms, from 1±0 to 12±2; n = 5, p<0.01) (Figure 4C). The hyperexcitability profile of compound 13 (1 µM) on spike generation was reflected by a decrease in rheobase current of about 300 pA that is needed to generate a single spike (for a 2 ms injection, from 650±71 pA to 347±44 pA, n = 6; p<0.01). The hyperexcitability discharge pattern induced by this M-channel blocker was so strong that in some cases, it could lead the DRG neurons to fire spontaneously, with no need of injecting depolarizing current (Figure 4D).

### M-channel opener properties of compound 15, an ester derivative that also inhibits COX enzymes

**Figure 5:**
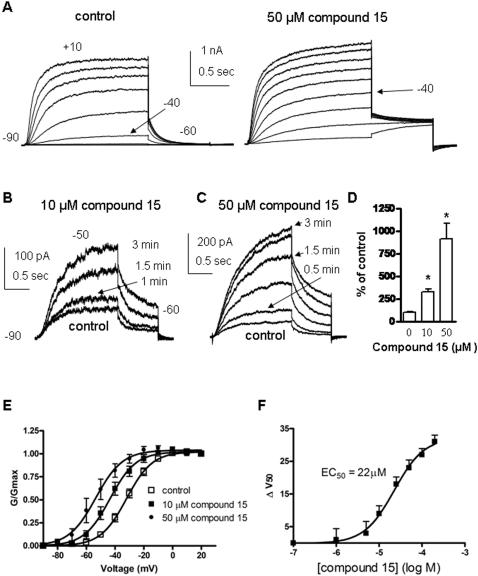
M-channel opener properties of compound 15 on Kv7.2/3 channels expressed in CHO cells. (A) Representative traces recorded from the same cell before (left panel) and after (right panel) external application 50 µM compound 15. The membrane potential was stepped from −90 mV (holding potential) to +10 mV for 1.5 s pulse duration in 10 mV increments, followed by a repolarizing step to −60 mV. (B) and (C) Cells were stepped from −90 mV to −50 mV every 30 sec for 1.5 sec pulse duration. Current traces were recorded from the same cell in the absence (control) and presence of 10 µM (B) and 50 µM (c) compound 15. (D) The percentage of the current recorded at −50 mV is shown in the presence of 10 µM and 50 µM compound 15 or in its absence, the latter being the control of 100% (n = 10; * p<0.01). (E) The normalized conductance (G/Gmax) was plotted as a function of the test voltages, for control (open squares), 10 µM (solid squares) and 50 µM (diamonds) compound 15-treated cells. The activation curves were fitted using one Boltzmann function (n = 5). (F) The potency of compound 15 was determined by the extent of left-shift (ΔV_50_), plotted as a function of compound 15 concentration and fitted by a sigmoidal function yielding an EC_50_ value of 22±1 µM (n = 5).

**Figure 6:**
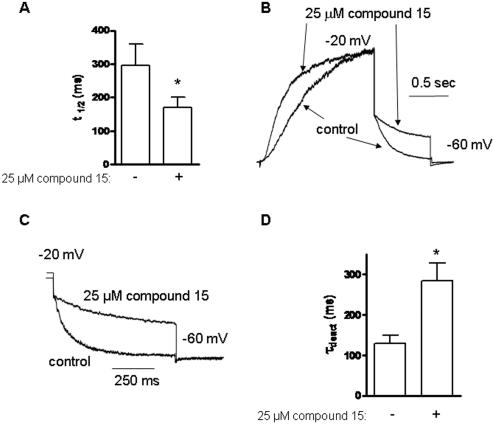
Effect of compound 15 on activation and deactivation kinetics of Kv7.2/3 channels. (A) Activation kinetics were evaluated at −20 mV by determining t_1/2_ , the time value at which half of the current amplitude developed, in the absence or presence of 25 µM compound 15 (n = 4; * p<0.02). (B) Representative normalized trace of current activation in the absence and presence of 25 µM compound 15. (C) Representative normalized trace of current deactivation at −60 mV in the absence and presence of 25 µM compound 15. (D) At −60 mV, deactivation kinetics were fitted by one exponential function and the time constant was measured in the absence and presence of 25 µM compound 15 (n = 4; * p<0.02).

As described above, we found that the ester compound 15 of the diclofenac series was both an inhibitor of the COX enzymes but also a potent opener of M-channels. We analyzed in more details its opener properties *in vitro* and *in vivo*. Figure 5A (left panel) shows representative traces of Kv7.2/3 channels expressed in CHO cells. External application of compound 15 enhanced Kv7.2/3 currents at nearly all voltages (right panel). However, like all openers described in this study, the effects of compound 15 were voltage-dependent. As the test potentials were more positive (above -10 mV), the effects of compound 15 became weaker on current amplitude. When membrane potential was stepped every 30 sec from −90 to −50 mV, application of 10 µM and 50 µM of compound 15 produced 3.2- and 9.1-fold increase in current amplitude, respectively (n = 10; Figure 5B–D). The drug action had a fast onset as within one minute of compound 15 superfusion, the current increased significantly. When we analyzed the conductance/voltage relationships, it was clear that the activating effect of compound 15 mainly arises from a left-shift in the Kv7.2/3 activation curve. For example, 10 µM, 50 µM and 200 µM of compound 15 respectively produced left-shifts of −11 mV, −21 mV and −31 mV compared to control, with no change in the Boltzmann slopes (Figure 5E and F; V_50_ = −32.1±8.9 mV, n = 19; V_50_ = −43.6±9.0 mV, n = 5; V_50_ = −53.5±9.3 mV, n = 5 and V_50_ = −63.1±2.0 mV, n = 5, respectively). The opener action of compound 15 was concentration-dependent and was quantified by the extent of the left-shift (ΔV_50_), yielding an EC_50_ of 22 µM. Compound 15 also affected Kv7.2/3 gating by accelerating the activation kinetics (from t_1/2_ = 296±63 ms to t_1/2_ = 171±29 ms, n = 6; p<0.05; Figure 6A and B). Comparison of the tail currents at −60 mV revealed that compound 15 markedly slowed down the deactivation kinetics of Kv7.2/3 channels (from τ_deact_ = 129±22 ms to τ_deact_ = 284±43 ms; n = 5, p<0.01; Figure 6C and D).

**Figure 7:**
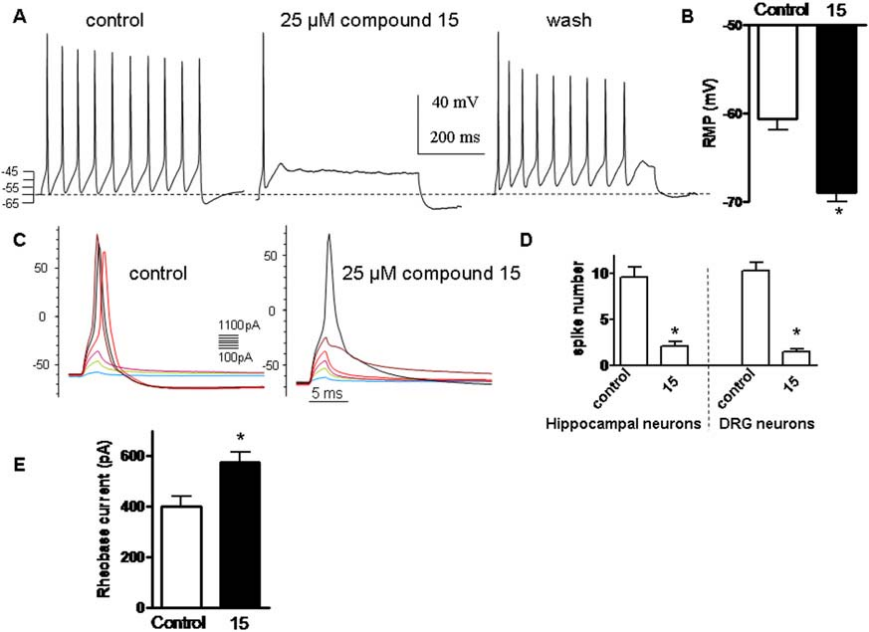
Compound 15 inhibits firing of hippocampal and peripheral DRG neurons. (A) Representative rat hippocampal spiking discharge, evoked by a squared depolarizing current pulse (100 pA for 400 msec) before (control), during exposure to 25 µM compound 15 and after washout (wash). (B) Resting membrane potential of DRG neurons before (control) and following exposure to 25 µM compound 15 (n = 13; * p<0.01). (C) Representative solitary spike evoked in DRG neurons by 2 ms squared depolarizing current pulses (100–1100 pA in 100 pA increments) in the absence (control) or presence of 25 µM compound 15. (D) Number of spikes evoked by injecting squared depolarizing current pulses (75–200 pA for 400 ms) in hippocampal and DRG neurons in the absence and presence of 25 µM compound 15 (n = 8; * p<0.01). (E) Rheobase current necessary to inject (2 ms) into DRG neurons to evoke a solitary spike in the absence and presence of 25 µM compound 15 (n = 12; * p<0.01).

We further characterized the properties of compound 15 on central and peripheral neurons, using the current-clamp mode of the whole-cell patch-clamp technique in rat primary cultures of hippocampal and DRG neurons (Figure 7). Repetitive spike discharges were evoked by depolarizing current injections. External application of compound 15 (25 µM) robustly and reversibly depressed the number of evoked action potentials in both hippocampal and DRG neurons (from 9.6±1.0 to 2.1±0.5, n = 8 and from 10.4±0.8 to 0.8±0.3, n = 8 respectively; 75–200 pA for 400 ms; p<0.01) (Figure 7A and D). Exposure to compound 15 (25 µM) hyperpolarized the resting membrane potential of DRG neurons by −8.3±0.2 mV (from −60.6±0.3 mV to −69.0±0.3 mV, n = 13; p<0.01; Figure 7B). A similar hyperpolarization was also obtained in hippocampal neurons exposed to compound 15 (−8.5 mV). Reflecting the dampening action of compound 15 on spike generation, the rheobase current needed to be injected into DRG neurons to generate a single spike, was increased upon exposure to the drug (for a 2 ms injection, from 400±41 pA to 577±38 pA, n = 12; p<0.01) (Figure 7C and E).

**Figure 8:**
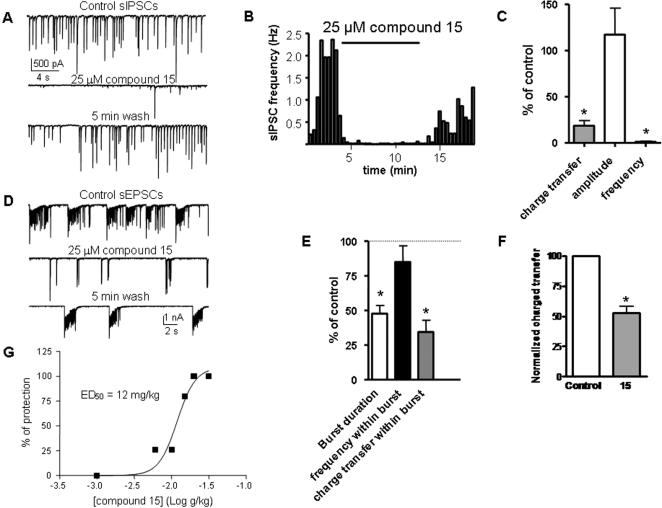
Effects of compound 15 on spontaneous glutamate and GABA release and on maximal electroshock seizure model in mice. (A) Representative traces of spontaneous IPSCs recorded at a holding potential of −70 mV, before (control), during exposure to 25 µM compound 15 and after washout (wash). (B) Representative experiment showing sIPSCs frequency as a function of time, before, during exposure to 25 µM compound 15 and after washout. (C) Effect of 25 µM compound 15 on normalized charge transfer, amplitude and frequency of sIPSCs (n = 7; * p<0.01). (D) Representative traces of spontaneous EPSCs recorded at a holding potential of −70 mV, before (control), during exposure to 25 µM compound 15 and after washout (wash). (E) effect of 25 µM compound 15 on normalized burst duration, on frequency and charge transfer within bursts and on total (F) charge transfer of sEPSCs (n = 6; * p<0.01). (G) Compound 15 protects from epileptic seizures induced by the MES generalized epilepsy model in mice (0.2 sec, 50 mA) with ED_50_ of 12 mg/kg.

Because of their slow activation and their lack of inactivation, M-channels could modulate neuronal activity during repetitive spike discharge and tune neurotransmitter release. Figure 8 illustrates the effect of compound 15 on synaptic transmission and transmitter release. Spontaneous excitatory and inhibitory post-synaptic currents (sEPSCs and sIPSCs) were recorded from dense cultures of primary hippocampal neurons, using the voltage-clamp configuration of the whole-cell patch-clamp technique. The sIPSCs were recorded at a holding potential of −70 mV and were pharmacologically isolated by blocking NMDA and AMPA receptor-mediated excitatory postsynaptic currents (with 10 µM AP-5 and 10 µM NBQX); sIPSCs were exclusively mediated by the activation of GABA_A_ Cl^−^ channels as they could be completely blocked with 30 µM picrotoxin and 10 µM bicuculline (not shown). Hence, sIPSCs reflect the synaptic release of GABA. Compound 15 (25 µM) powerfully and reversibly inhibited sIPSCs, mainly by depressing their frequency of spontaneous occurrence (more than 98% inhibition, Figure 8A–C). Compound 15 did not significantly affect the amplitude and the kinetics of the currents. Spontaneous EPSCs were recorded at −70 mV and were pharmacologically isolated by blocking GABA_A_ receptor-mediated inhibitory post-synaptic currents (with 10 µM bicuculline and 30 µM picrotoxin). The sEPSCs were solely mediated by the activation of AMPA receptors and to a lesser extent of NMDA receptors, as they were blocked by 10 µM NBQX and 10 µM AP-5. Thus, sEPSCs reflect the synaptic release of glutamate. Under control conditions, sEPSCs appeared as repetitive bursts whose frequency was variable, ranging from 0.3 to 2 Hz and increasing with the density of the hippocampal culture. Application of compound 15 markedly reduced the burst duration of sEPSCs without a significant effect on the amplitude (Figure 8D–F). Consequently, compound 15 reduced by about 50% the total charge transfer as well as the charge transfer within the bursts (Figure 8D–F). Interestingly, activation of M-channels by compound 15 did not affect the amplitude and the kinetics of miniature EPSCs and IPSCs (not shown).

Given its dampening action on neuronal spiking discharges, we checked for a possible anti-convulsant effect of compound 15 in the maximal electroshock seizure (MES) model in mice. This test is generally thought to be a model of generalized tonic-clonic seizure in human [19]. MES produced hind limb extension in all mice that received intraperitoneal injection of vehicle solution. Intraperitoneal injection of compound 15 30 min before the electroshock, dose-dependently (1–40 mg/kg) protected the animals against the tonic extension caused by MES, with an ED50 of 12 mg/kg (Figure 8G). For comparison, intraperitoneal injection of phenytoin and sodium valproate 30 min before the electroshock fully prevented hind limb extension at doses of 20 mg/kg and 500 mg/kg, respectively (not shown).

### M-channel opener properties of compound 6, an amide derivative that does not inhibit COX enzymes

**Figure 9:**
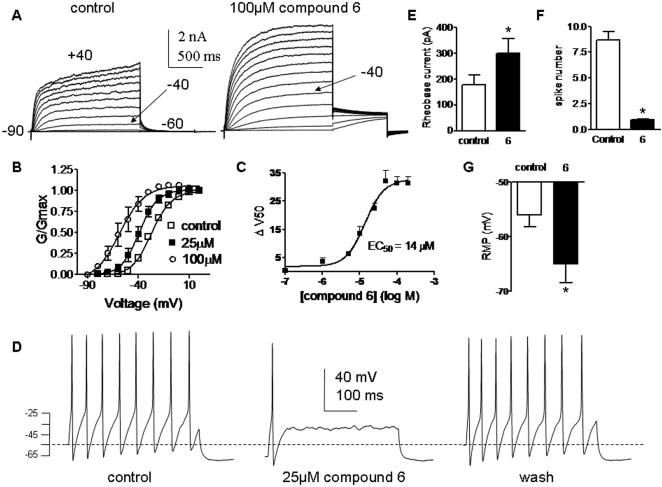
Compound 6 enhances Kv7.2/3 currents and inhibits firing of peripheral DRG neurons. (A) Representative traces recorded from the same CHO cell before (left panel) and after (right panel) external application 100 µM compound 6. The membrane potential was stepped from −90 mV (holding potential) to +40 mV for 1.5 s pulse duration in 10mV increments, followed by a repolarizing step to −60 mV. (B) The normalized conductance (G/Gmax) was plotted as a function of the test voltages, for control (open squares), 25 µM (solid squares) and 100 µM (empty circles) compound 6-treated cells. The activation curves were fitted using one Boltzmann function (n = 5). (C) The potency of compound 6 was determined by the extent of left-shift (ΔV_50_), plotted as a function of compound 6 concentration and fitted by a sigmoidal function yielding an EC_50_ value of 14±2 µM (n = 5). (D) Representative rat DRG spiking discharge, evoked by a squared depolarizing current pulse (100 pA for 400 msec) before (control), during exposure to 25 µM compound 6 and after washout (wash). (E) Rheobase current necessary to inject (2 ms) into DRG neurons to evoke a solitary spike in the absence and presence of 25 µM compound 6 (n = 8; * p<0.01). (F) Number of spikes evoked by injecting squared depolarizing current pulses (75–200 pA for 400 ms) in DRG neurons in the absence and presence of 25 µM compound 6 (n = 12; * p<0.01). (G) Resting membrane potential of DRG neurons before (control) and following exposure to 25 µM compound 6 (n = 7; * p<0.01).

As mentioned above, we succeeded to design an amide derivative of the diclofenac series, which displays a potent M-channel activity but does not inhibit COX-1/COX-2 enzymes. We characterized further its action on recombinant Kv7.2/3 channels expressed in CHO cells and its properties on primary cultured neurons. Superfusion of compound 6 enhanced Kv7.2/3 currents at all voltages. Like compound 15, the activating effects of compound 6 became weaker when membrane voltage was stepped to more positive potentials (Figure 9A). At −50 mV, a physiologically relevant subthreshold potential, compound 6 (25 µM) increased Kv7.2/3 current amplitude by 9.4±1.2 fold (n = 5). The activating effect of compound 6 mostly originated from a left-shift of the Kv7.2/3 activation curve. For example, 10 µM and 100 µM of compound 6 produced left-shifts of −13 mV and −31 mV, respectively, compared to control (Figure 9B and C). Compound 6 dose-dependently activated Kv7.2/3 channels, a feature quantified by the concentration dependent left-shift (ΔV_50_), yielding an EC_50_ of 14 µM (Figure 9C).

We examined the selectivity of compound 6 on other relevant Kv channels expressed in *Xenopus* oocytes (Table 2). Except for a moderate inhibition of Kv1.2 and Kv7.1 currents (34% and 23 %, respectively), compound 6 (50 µM) did not affect the current amplitude of Kv11.1 (hERG), Kv2.1 and Kv4.3. Among the Kv7 channel family, compound 6 appeared to act as an opener of Kv7.2 but not of Kv7.1 and Kv7.4. We found that compound 6 could also activate Kv7.3 when tested on the Kv7.3 mutant A315T [20]. Noteworthy, compound 6 (25 µM) did not affect the amplitude of miniature excitatory and inhibitory post-synaptic currents (mEPSCs and mIPSCs) in cultured hippocampal neurons, suggesting that it does not interact with AMPA/NMDA and GABA_A_ channels (not shown).

**Table 2:** Specificity of compound 6 on different K^+^ channel subtypes.

K^+^ channel subtype	Fold current amplitude	
	25 µM compound 6	50 µM compound 6
Kv11.1 or hERG (−10 mV)	1.1±0.02	0.9±0.08
Kv1.2 (0 mV)	0.9±0.01	0.66±0.03 *
Kv 2.1 (+30 mV)	1.2±0.09	1.2±0.11
Kv 4.3 (+30 mV)	1.1±0.04	1.0±0.07
Kv7.2 (−40 mV)	3.5±0.4 *	6.8±0.7 *
Kv 7.4 (−30 mV)	1.0±0.12	0.9±0.04
Kv 7.1 (+30 mV)	0.93±0.02	0.72±0.02 *
I_KS _(+30 mV)	1.02±0.04	0.97±0.04

The specificity of compound 6 at 25 µM and 50 µM towards various Kv channels was tested in *Xenopus* oocytes, except for Kv1.2 which was checked in transfected CHO cells. The current amplitude of the various Kv channels was tested at the indicated voltages and the effect of the drugs was expressed as fold of the control amplitude measured under the same conditions in the absence of the drug. Data are expressed as mean±SEM of 5-7 separate experiments. * significance p<0.01.

Superfusion of compound 6 (25 µM) powerfully and reversibly reduced the number of evoked action potentials in DRG neurons (from 8.7±0.8 to 0.9±0.1, n = 12; 75–250 pA for 400 ms; p<0.001) (Figure 9D and F). Exposure to compound 6 hyperpolarized the resting membrane potential of DRG neurons by −9.0±1.9 mV (from −56.0±2.2 mV to −65.0±3.4 mV, n = 7; p<0.01; Figure 9G). The rheobase current needed to generate a single spike in DRG neurons was increased upon exposure to compound 6 (for a 2 ms injection, from 178±36 pA to 300±55 pA, n = 8; p<0.005) (Figure 9E).

## Discussion

Based on carboxylate derivatization into amides and esters, our data reveals a crucial determinant of COX inhibition by the diclofenac and meclofenamate NSAID subfamilies. The SAR study indicates that the conversion of the carboxylate group to amide functionality efficiently dissociates the COX inhibition from the M-channel modulation, generating M-channel openers free of significant anti-COX activity. This functional switch is clearly observed with the amide derivatives of the diclofenac series, compounds 1, 2, 4, 6 and 7, which lack substantial COX enzyme inhibition but are all M-channel openers, provided that a terminal hydroxyl function is maintained (Figure 2, see below). This feature contrasts with the ester derivatives of the same diclofenac series, compounds 8, 9 and 15, which are powerful COX inhibitors as well as M-channel openers. The same characteristics are observed with the amide derivatives of the meclofenamate series such as compounds 16–18. In previous SAR studies, attempts were made for derivatizing the carboxylate moiety of various NSAIDs to esters and amides in order to produce selective COX-2 inhibitors or antimycobacterial agents [21]–[25]. For example, diclofenac amide derivatives were recently found to exhibit enhanced antimycobacterial activity [25]. Esters, primary and secondary amides of indomethacin were shown to be selective COX-2 inhibitors [22], [23]. Unlike the indomethacin series, wherein both esters and amides are potent and selective COX-2 inhibitors, SAR studies on meclofenamic acid analogues suggest that only some amide derivatives are capable of COX-2-selective inhibition [22], [24]. However, it was found that introduction of a terminal hydroxyl group in the alkyl chain leads to significant loss in potency and selectivity [22], [24]. Interestingly, all amide compounds characterized in this work possess a terminal hydroxyl moiety. Hence, the combination of the amide function and the preservation of a terminal hydroxyl group are probably responsible for the profound loss of COX inhibition displayed by the amide derivatives, designed in this study.

The active site of COX-1 and COX-2 isozymes is a narrow hydrophobic channel extending from the membrane binding domain to the core of the catalytic domain [1], [26]. In the apex of the channel, both enzymes possess a conserved Ser530 corresponding to the residue acetylated by aspirin and a conserved Tyr385, which is involved in the hydroperoxidase activity. Two conserved charged residues, Arg120 and Glu524, are also located at the entrance of the COX active site. Many NSAIDs bearing a carboxylate function are known to form a salt bridge with Arg120 and to engage into hydrogen bonding with the phenolic hydroxyl of Tyr355. Crystal structures of the COX enzymes with several carboxylic acid-containing NSAIDs show that the inhibitors have their carboxylate coordinated to Arg120 and their aromatic functionality projecting into the cyclooxygenase active site toward Tyr385 [27], [28]. For example, recent molecular docking studies indicate that the carboxylates of flurbiprofen, ketoprofen and naproxen form a salt bridge with Arg120 and a hydrogen bond with the phenolic hydroxyl of Tyr355 [29]. The three inhibitors bind to the same site and adopt similar conformations to those found in the crystal structures of ovine COX-1 complexed with ibuprofen and flurbiprofen [30]. In fact, the carboxylate moiety can be in ortho, meta or para positions with respect to the aromatic moiety of the molecule. Notably, this seems to make a difference in the binding mode of the ligands. When the carboxylate moiety is in meta or para, the inhibitor binds in such a way that the carboxylate forms a salt bridge with Arg120. In contrast, when the carboxylate moiety is in ortho, ligands like diclofenac or meclofenamic acid bind in a different orientation. The recent determination of the crystal structure of diclofenac complexed with COX-2 demonstrates that diclofenac binds to COX-2 in an inverted conformation with its carboxylate group being hydrogen-bonded to Tyr-385 and Ser-530, a feature consistent with the structure of the complex arachidonic acid-apoCOX-2 [31]. In addition, the phenyl group linked to the acetic acid moiety of diclofenac was shown to form extensive van der Waals interactions with several hydrophobic residues within the active site [31].

How might carboxylate conversion into amide affect the COX inhibition? The absence of a carboxylate group in compounds of this study suggests that they probably interact differently from diclofenac. It is likely that the amide bond is stiffer than the ester bond, thus leading to a reduced flexibility of the CO-NH**-C-C** bond, compared to the CO-O-**C-C** bond. If so, the flexibility of the entire alkyl/ether chain would be altered, thus restricting the number of chain conformations that could accommodate into the active site pocket of the COX enzyme, mainly because of the rigidity of this moiety. Interestingly, recent molecular dynamics studies indicate that diclofenac and meclofenamic acid binding modes need to occupy a larger region in the neighborhood of Ser530 that is not required by other NSAIDs [32]. Similar features have been previously observed for diphenylacetyl derivatives of acetylcholine where replacement of ester by amide increases the stiffness of the **C-C** bond, reduces the proportion of ‘gauche’ conformer and decreases the affinity for muscarinic acetylcholine receptors [33]. In addition, esters are hydrogen-bond acceptors, but cannot act as hydrogen-bond donors. Hence, Tyr-385 and Ser-530 may form hydrogen bonds with the ester function instead of the carboxylate of diclofenac, making the ester derivatives potent COX inhibitors. In contrast, secondary amides can act as hydrogen-bond donors and acceptors and may thus interact via a very different hydrogen bonding network.

Considering the properties of the compounds pertaining to the M-channel activity, several contrasting features could be observed when one compares them with those required for the COX inhibition. First, the derivatization of the carboxylate into amide or ester is not important for displaying M-channel properties, in striking contrast to its impact on COX enzyme inhibition. This is clearly exemplified by compounds 8 and 2, which vis-à-vis the M-channel target, can accommodate with ester and amide functions, respectively, since both are openers, while only ester compound 8 inhibits COX activity. Second, the bridging secondary amine of the diphenylamine moiety of diclofenac and meclofenamate is not important for COX inhibition and is not engaged in any close interactions with residues of the COX active site, as previously shown in crystallographic data [31]. In contrast, we found that compounds lacking this secondary amine and still bearing a carboxylate (e.g., flurbiprofen, ketoprofen or fenoprofen) do not activate Kv7.2/3 channels. This implies that the bridging secondary amine function crucially interacts with an electrophile or is engaged into hydrogen bonding within a hydrophobic pocket site of the M-channel. Along this line, we found that aromatic ring deactivators (electron-withdrawing groups) like NO_2_ may increase the opener potency as shown by comparing compounds 2 or 17 with compound 6 (Table 1 and Table S1). Third, our data indicate that a terminal hydroxyl group in the ether and alkyl chain is absolutely required to generate an M-channel opener (Figure 2). This functionality has two reactive covalent bonds, the C–O and the O–H bonds, which are polarized so that oxygen is electron rich and may potentially react with an electrophile while hydrogen may be involved in hydrogen bonding. When the terminal hydroxyl function has been removed, none of the derivatives obtained are M-channel openers as exemplified by compounds 10, 12, 19 and 20. Remarkably, the crucial role of this terminal hydroxyl function is further underscored when it is replaced by isobutyl, ethylamine or 2,3-epoxypropyl groups in derivatives of the diclofenac series, which generate M-channel blockers. This unique feature is best illustrated by compound 13 which is a potent M-channel blocker, yet based on the very same template, thus suggesting that the opener and blocker molecules probably bind to the same channel site. Indeed, the tiny change of a hydroxyl group to amine functionality generates a compound that potently inhibits recombinant Kv7.2/3 channels and induces a hyperexcitability state in DRG neurons, which fire spontaneously upon compound 13 exposure. The dual function of M-channel blocker and COX inhibitor exemplified by compound 13 may be of great therapeutic advantage for treating memory deficits and Alzheimer's disease. Indeed, administration of NSAID agents is known to reduce the risk of developing Alzheimer's disease in normal aging populations [34], an effect that may be reinforced by the cognitive enhancer action obtained upon blockade of M-channels [14]. Similarly, the dual function of compounds 8 or 15, as COX inhibitors and M-channel openers, may produce synergistic effects in terms of pain management. Thus, it may be potentially useful in some cases to exploit the dual function of some derivatives to reinforce their therapeutic value.

The good selectivity of the opener compound 6 for M-channels *versus* other Kv channels, including the cardiac Kv11.1 (hERG or *I_KR_*) and Kv7.1/KCNE1 (*I_KS_*) and other neuronal K^+^ currents, is promising. *I_KR_* and *I_KS_* currents are essential for the normal repolarization of the cardiac action potential and mutations of their encoding genes produce the long QT syndrome (LQT), a human ventricular arrhythmia [35]. Common medications (antihistamines, antiarrhythmics, antibiotics) can block the Kv11.1 (hERG) channel and thereby depress the cardiac *I_KR_* current [36]. This side effect leads to similar risks of lethal arrhythmia as those produced by the inherited LQT syndrome. The lack of blocking effects of compound 6 on *I_KR_* and *I_KS_* currents opens interesting therapeutic perspectives for these new derivatives. Noteworthy, compound 6 is selective for Kv7.2/3 channels and does not activate Kv7.4, which is a distinctive feature compared to that of the well known opener retigabine [37].

In all, this study reveals important determinants of COX inhibition by the NSAID subfamilies of diclofenac and meclofenamic acid. The data also provide a structural framework for tailoring powerful and selective M-channel modulators, including openers and blockers. In addition, this study offers premises for designing new NSAIDs.

## Methods

### Synthesis of compounds

All details concerning the synthesis of compounds can be found in the Supporting Text S1.

### Recombinant expression of Kv7.2/3 channels in CHO cells

Chinese hamster ovary (CHO) cells were grown in Dulbecco's modified Eagle's medium (DMEM) supplemented with 2 mM glutamine, 10% fetal calf serum and antibiotics. Briefly, 40,000 cells seeded on poly-D-lysine-coated glass coverslips (13 mm diameter) in a 24-multiwell plate were transfected with pIRES-CD8 (0.5 µg) as a marker for transfection, and with Kv7.2 (0.5 µg) and Kv7.3 (0.5 µg). For electrophysiology, transfected cells were visualized approximately 40 hours following transfection, using anti-CD8 antibody-coated beads. Transfection was performed using Fugene 6 (Roche, Indianapolis IN, USA) according to the manufacturer's protocol.

### Neuronal hippocampal and DRG primary cultures

For dorsal root ganglion (DRG) neuronal cultures, ganglia were dissected from 2–5 day-old Sprague-Dawley rats killed by decapitation. DRGs were placed in Hank's balanced saline solution (HBSS) and prepared by enzymatic dissociation. Briefly, after 30 min incubation in 5 mg/ml dispase, 2 mg/ml collagenase type 1A and 0.1 mg/ml DNase (Invitrogen/Gibco, Carlsbad, CA) in Ca^2+^ and Mg^2+^-free HBSS, the ganglia were mechanically triturated with a fire-polished glass Pasteur pipette. The ganglia were then centrifuged for 5 min at 80xg and resuspended in DMEM supplemented with 2 mM L-glutamine, 16.5 mM NaHCO_3_, 6 g/l glucose, 5 ml penicillin/streptomycin and 10% fetal calf serum. For electrophysiological recording, dissociated neurons were plated on 13 mm glass coverslips, previously coated with poly-D-lysine (1 mg/ml) and laminin (10 µg/ml) and used at 1–2 days in culture.

For hippocampal neuronal cultures, Sprague-Dawley rat embryos (E18) were removed by caesarian section and their hippocampi were dissected out. The tissue was digested with papain for 20 min, triturated to a single-cell suspension, and plated at a density of 40,000 cells per ml on a substrate of bovine collagen type IV and 100 µg/ml poly-L-lysine in 13 mm diameter glass coverslips of a 24-multiwell plate. The culture medium consisted of Modified Eagle's Medium containing 5% horse serum (Biological Industries, Beit HaEmek, Israel), B-27 neuronal supplement (Invitrogen, Carlsbad, CA), 100 U/ml penicillin, 100 µg/ml streptomycin, and 2 mM glutamine. D-glucose was supplemented to a final concentration of 6 g/l. Cytosine-1-D-arabinofuranoside (5 µM) was added after 5 days to arrest glial cell proliferation. For electrophysiological recordings hippocampal neurons were used at 10–14 days in culture. All cultures were maintained at 37°C in humidified air containing 5% CO_2_.

### Maximal Electroshock seizure test

The anti-convulsant effect was measured by the maximal electroshock seizure model (MES) in ICR mice as previously described [7]. The procedures followed for experimentation and maintenance of the animals were approved by the animal research ethics committee of Tel Aviv University and in accordance with the Guide for the Care and Use of Laboratory Animals (1996, National Academy of Sciences, Washington DC). Briefly, maximal electroshock was induced in adult mice by means of two transcorneal electrodes delivering an alternative current of 50 mA at 60 Hz for 0.2 sec using rodent shocker (Hugo Sachs Electronik, type 221). This was shown to cause tonic convulsions in 100% of the animals tested. The drugs dissolved in 0.9% saline were administered intraperitoneally 30 min before the electroshock was performed. Animals failing to show tonic hind limb extension were scored as protected and were expressed in percentage.

### Electrophysiology

Voltage-clamp recordings in CHO cells were performed, using the whole-cell configuration of the patch-clamp technique. Signals were amplified using an Axopatch 200B patch-clamp amplifier (Molecular Devices, Sunnyvale, CA), sampled at 2 kHz and filtered at 800 Hz via a 4-pole Bessel low pass filter. Data were acquired using pClamp 9.2 software (Molecular Devices) and an IBM compatible Pentium IV computer in conjunction with a DigiData 1322A interface (Molecular Devices). The patch pipettes were pulled from borosilicate glass (Warner Instrument Corp, USA) with a resistance of 2–5 MΩ. For K^+^ current recordings in CHO cells, the intracellular pipette solution contained (in mM): 130 KCl, 1 MgCl_2_, 5 K_2_ATP, 5 EGTA, 10 HEPES, adjusted with KOH to pH 7.4 (290 mOsm). The extracellular solution contained (in mM): 140 NaCl, 4 KCl, 1.8 CaCl_2_, 1.2 MgCl_2_, 11 glucose, 5.5 HEPES, adjusted with NaOH to pH 7.4 (310 mOsm). Series resistances (3–13 MΩ) were compensated (75–90%) and periodically monitored.

For current-clamp recordings in primary cultured neurons, the patch pipettes were filled with (in mM): 135 KCl, 1 K_2_ATP, 1 MgATP, 2 EGTA, 1.1 CaCl_2_, 5 glucose, 10 HEPES, adjusted with KOH at pH 7.4 (315 mOsm). The external solution contained (in mM): 150 NaCl, 2.5 KCl, 2 CaCl_2_, 2 MgCl_2_, 15 glucose, 10 HEPES, adjusted with NaOH at pH 7.4 (325 mOsm). Recordings were sampled at 5 kHz and filtered at 2 KHz via a 4-pole Bessel low pass filter. A liquid junction potential of about −15.6 mV was measured between the intracellular and saline solutions and corrected on-line. For evoking spike discharges, 75–200 pA square depolarizing current pulses were injected into neurons for 400 ms. For rheobase current measurements, 100–1100 pA square depolarizing current pulses were injected into neurons for 2 ms.

Spontaneous excitatory postsynaptic currents (EPSCs) were recorded in the voltage-clamp configuration of the whole-cell patch-clamp technique at a holding potential of −70 mV as previously described [38]; the extracellular solution contained (in mM) 160 NaCl, 2.5 KCl, 10 HEPES, 10 glucose, 2 CaCl_2_; pH 7.3 (325 mOsm), to which 30 µM picrotoxin and 10 µM bicuculline methyl iodide were added. The intracellular solution consisted (in mM) of 130 K-gluconate, 10 KCl, 1.1 EGTA, 10 HEPES, 1 MgCl_2_, 2 Na_2_ATP, 0.1 CaCl_2_ and 5 QX314Br to block Na^+^ currents; pH 7.2 (315 mOsm). Spontaneous inhibitory postsynaptic currents (IPSCs) were recorded at a holding potential of −70 mV; the extracellular solution contained (in mM) 160 NaCl, 2.5 KCl, 10 HEPES, 10 glucose, 2 CaCl_2_; pH 7.3 (325 mOsm), to which 10 µM NBQX and 10 µM AP-5 were added. The intracellular solution consisted (in mM) of 144 CsCl, 10 HEPES, 1.1 EGTA, 0.1 CaCl_2_, 5 MgCl_2_, 2 Na_2_ATP, 5 QX314 Br; pH 7.3 (315 mOsm).

Voltage-clamp measurements in *Xenopus* oocytes were performed as previously described [7].

### Data analyses

Data analysis was performed using the Clampfit program (pClamp 9.2, Molecular Devices), Microsoft Excel (Microsoft) and Prism 4.0 (GraphPad). Chord conductance (G) was calculated by using the following equation:
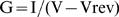
where I corresponds to the current amplitude measured at the end of the pulse and Vrev, the calculated reversal potential. G was estimated at various test voltages V and then, normalized to a maximal conductance value, Gmax. Activation curves were fitted by one Boltzmann distribution:
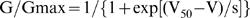
where V_50_ is the voltage at which the current is half-activated and s is the slope factor. All data were expressed as mean±SEM. Statistically significant differences were assessed by Student's t-test. Analysis of sEPSCs and sIPSCs was done using the Clampfit program (pClamp 9.2, Molecular Devices) and included evaluation of the individual events as well as the average event amplitude and the integral of the event (total charge transfer).

### Cyclooxygenase inhibition assays

Mouse colon adenocarcinoma CT26 and mouse Lewis lung carcinoma D122 cells were grown in DMEM (D122) or RPMI (CT26) supplemented with 10% fetal calf serum, 4% glutamine, 1% penicillin-streptomycin-nystatin and 3% non essential amino acids (D122) and maintained in a humidified 37°C incubator with 5% CO2. For the COX assays, cells were grown onto 24 multiwell plates. Before the assay, cells were washed with serum-free medium; then tested compounds were added to the medium and incubated for 30 min at 37°C after which 30 µM arachidonic acid was added and incubated for additional 20 min. At the end of the incubations, 10 µM indomethacin was added to stop the reaction and medium was transferred to another 24 multiwell plate for measuring PGE2 production by radioimmunoassay (DuPont-NEN; Boston, MA) according to manufacturer protocol. Protein determination was assayed on scraped cells and was used to normalize the amount of PGE2 production. Assays with purified ovine COX-1 and human recombinant COX-2 were performed using the COX inhibitor screening assay (Cayman chemical company, Ann Arbor, MI), according to manufacturer protocol.

## Supporting Information

Table S1 (0.06 MB DOC)

Text S1 (0.21 MB DOC)
