# Supplementary material for: A Tale of Switched Functions: From Cyclooxygenase Inhibition to M-Channel Modulation in New Diphenylamine Derivatives
Source: PLoS One. 2007 Dec 26;2(12):e1332. doi: 10.1371/journal.pone.0001332 (PMC2131780; doi:10.1371/journal.pone.0001332)
Supplement: Table S1 — (0.06 MB DOC) [file pone.0001332.s001.doc]

**Table S1. Structures of derivatives of diclofenac and meclofenamic acid**

**--------------------------------------------------------------------------------**

**Compound R1 A1 A2 A3**

Diclofenac OH H H H

1 H H H

2 H H H

3 OH NO_2_ H H

4 H H H

5 NO_2_ H NO_2_

6 NO_2_ NO_2_ NO_2_

7 NO_2_ H H

8 H H H

9 H H H

10 H H H

11 H H H

12 H H H

13 H H H

14 H H H

15 NO_2_ H H

**Compound** **R2 B1 B2 B3**

Meclofenamic acid OH Cl Cl CH3

16 CH3 H CH3

17  H H CF3

18 CH3 H Cl

19 CH3 H Cl

20 H H CF3
