# Supplementary material for: A Tale of Switched Functions: From Cyclooxygenase Inhibition to M-Channel Modulation in New Diphenylamine Derivatives
Source: PLoS One. 2007 Dec 26;2(12):e1332. doi: 10.1371/journal.pone.0001332 (PMC2131780; doi:10.1371/journal.pone.0001332)
Supplement: Text S1 — (0.21 MB DOC) [file pone.0001332.s002.doc]

**Supporting Text S1**

**Synthesis of compounds**

**Abbreviations.** Boc- t-butoxycarbonyl, DCM- Dichloromethane, DMAP- Dimethylaminopyridine, DMF- Dimethyl formamide, DCC- Dicyclohexylcarbodiimide, EtOAc- Ethylacetate, He- Hexanes, RT- Room temprature, , TFA- Trifluoroacetic acid.

**General**: All reactions requiring anhydrous conditions were performed in oven-dried glassware under an Ar or N_2_ atmosphere. Chemicals and solvents were either A.R. grade or purified by standard techniques. Thin layer chromatography (TLC): silicagel plates Merck 60 F_254_; silicagel preparative plates Analtech 1000 microns; compounds were visualized by irradiation with UV light and/or by treatment with a solution of 25 g phosphomolybdic acid, 10 g Ce(SO_4_)_2_·H_2_O, 60 ml conc. H_2_SO_4_ and 940 ml H_2_O, followed by heating and/or by staining with a solution of 12 g 2,4-dinitrophenylhydrazine in 60 ml conc. H_2_SO_4_; 80 ml H_2_O and 200 ml 95% EtOH, followed by heating and/or immersing into an iodine bath (30 g I_2_, 2 g KI, in 400 ml EtOH/H_2_O 1:1) and warming. Flash chromatography (FC): silica gel Merck 60 (partical size 0.040-0.063 mm), eluent given in parentheses. 1H NMR: Bruker AMX 200 or 400. The chemical shifts are expressed in δ relative to TMS (δ = 0 ppm) and the coupling constants *J* in Hz. The spectra were recorded in CDCl_3_ or CD3OD as a solvent at room temperature unless stated otherwise. All reagents, including salts and solvents, were purchased from Aldrich (Milwaukee, MN). Compound **3** was synthesized according to reference 2.

**(a) General procedure for preparation of amide.**

N-Hydroxysuccinimide (0.76mmol) and DCC (0.76 mmol) were added to the corresponding acid (0.506 mmol) dissolved in 10 ml of dichloromethane. The mixture was stirred for 1 hour and monitored by TLC (EtOAc:He=1:1). Upon completion of the reaction, the mixture filtered and the solvent was removed under reduced pressure. The residue was dissolved in DMF. Then, the corresponding amine (0.6 mmol) was added. The mixture was stirred for 10 min at RT and monitored by TLC (EtOAc:Hex = 3:1). After completion the reaction was diluted with EtOAc and washed with ammonium chloride saturated solution. The organic layer was dried over magnesium sulfate and the solvent was removed under reduced pressure. The crude product was purified by column chromatography on silica gel to give the compounds **1, 2, 16, 17** and **18**.

**Compound 1.** This compound was synthesized as described in procedure (a). Yield 63%. RP-HPLC assay, C-18 analytical column, Wavelength; 275 nm, Eluent; acetonitrile:water using gradient elution 0-20 minutes starting with 10% to 100% acetonitrile, 20-25 minutes 100% acetonitrile, Flow rate; 1 ml/min, R.T; 8.34 min, Purity >95%. ^1^HNMR (200 MHz, CDCl_3_): δ=7.35 ppm (2H, d, J = 8 Hz); 7.19-7.06 (2H, m); 6.99 (1H, d, J = 8 Hz); 6.96 (1H, d, J = 8 Hz); 6.56 (1H, d, J = 8 Hz); 3.78-3.74 (2H, m); 3.64-3.57 (2H, m); 3.23 (2H, s); 2.95 (3H, s). ^13^C(400MHz, CDCl_3_) δ=174.0, 144.3, 138.7, 131.4, 130.7, 129.8, 128.3, 126.1, 125.0, 121.1, 118.5, 62.1, 52.6, 38.8 , 34.7. HRMS (MALDI-TOF): calculated for C_17_H_18_Cl_2_N_2_O_2_ 375.0637 [M+Na^+^], found 375.0608.

**Compound 2**. This compound was synthesized as described in procedure (a). Yield 78%. RP-HPLC assay, C-18 analytical column, Wavelength; 275 nm, Eluent; acetonitrile:water using gradient elution 0-20 minutes starting with 10% to 100% acetonitrile, 20-25 minutes 100% acetonitrile, Flow rate; 1 ml/min; R.T, 8.88 min, Purity >95%. ^1^HNMR (200MHz, CDCl_3_) δ=7.35 ppm (2H, d, J = 8 Hz); 7.19-7.06 (2H, m); 6.99 (1H, d, J = 8 Hz); 6.96 (1H, d, J = 8 Hz); 6.56 (1H, d, J = 8 Hz); 3.73-3.67 (4H, m); 3.57-3.47(6H, m). ^13^C(400MHz, CDCl_3_) δ=172.6, 143.7, 138.3, 131.4, 130.8, 129.6, 128.7, 125.3, 125.0, 122.2, 118.1, 73.0, 70.5, 62.5, 41.7, 40.3. HRMS (MALDI-TOF): calculated for C_18_H_20_Cl_2_N_2_O_3_ 383.0924 [M+Na^+^], found 383.0923.

**Compound 16.** This compound was synthesized as described in procedure (a). Yield 88%. RP-HPLC assay, C-18 analytical column, Wavelength; 285 nm, Eluent; acetonitrile:water using isocart elution with 12% acetonitrile, Flow rate; 1 ml/min, R.T; 6.47 min, Purity >95%. ^1^HNMR (200 MHz, CDCl_3_): δ = 7.43 (1H, dd, J = 2, J = 8 Hz); 7.21-7.14 (2H, m); 7.06 (1H, t, J = 8 Hz); 6.96-6.93 (2H, m); 6.67 (1H, d, J = 8 Hz); 3.6-3.8 (8H, m); 2.32 (3H, s); 2.20 (3H, s). ^13^C(400MHz, CDCl_3_) δ=170.6, 147.9, 140.3, 138.8, 133.0, 131.6, 129.5, 128.2, 126.5, 126.3, 121.6, 117.5, 115.6, 73.0, 70.7, 62.5, 40.4, 21.4, 14.6. HRMS (MALDI-TOF): calculated for C_19_H_24_N_2_O_3_ 351.1679 [M+Na^+^], found 351.1683.

**Compound 17.** This compound was synthesized as described in procedure (a). Yield 79%. RP-HPLC assay, C-18 analytical column, Wavelength; 290 nm, Eluent; acetonitrile:water using isocart elution with 12% acetonitrile, Flow rate; 1 ml/min, R.T; 5.87 min, Purity >95%. ^1^HNMR (200 MHz, CDCl_3_): δ = 7.48 (1H, dd, J = 2, J = 8 Hz); 7.4 (2H, d, J = 8 Hz); 7.32-7.16 (3H, m); 7.09 (1H, bs); 6.82 (1H, dt, J = 2, J = 8); 3.74-3.54 (8H, m). ^13^C(400MHz, CDCl_3_) δ=170.2, 144.9, 143.1, 133.0, 132.3, 130.6, 128.6, 126.2, 123.5, 120.2, 119.9, 119.0, 116.8, 116.6, 73.0, 70.5, 62.5, 40.3. HRMS (MALDI-TOF): calculated for C_18_H_19_F_3_N_2_O_3_ 391.1240 [M+Na^+^], found 391.1239.

**Compound 18.** This compound was synthesized as described in procedure (a). Yield 86%. RP-HPLC assay, C-18 analytical column, Wavelength; 289 nm, Eluent; acetonitrile:water using isocart elution with 12% acetonitrile, Flow rate; 1 ml/min, R.T; 6.14 min, Purity >95%. ^1^HNMR (200 MHz, CDCl_3_): δ = 7.47 (1H, dd, J = 2, J = 8 Hz); 7.22-6.99 (5H, m); 6.65 (1H, dt, J = 2, J = 8 Hz); 3.74-3.54 (8H, m); 2.33 (3H, s). ^13^C(400MHz, CDCl_3_) δ=170.4, 146.9, 142.0, 136.2, 133.1, 130.6, 128.3, 127.4, 125.0, 121.1, 118.5, 118.3, 116.0, 73.0, 70.6, 62.6, 40.3 ,15.2. HRMS (MALDI-TOF): calculated for C_18_H_21_ClN_2_O_3_ 371.1133 [M+Na^+^], found 371.1164.

Compound 4

Diclofenac (600 mg, 2 mmol) was dissolved in DCM. Then, N-Hydroxysuccinimide (260 mg, 2.2 mmol) and DMAP (16 mg, 0.14 mmol) were added. To this mixture DCC (500 mg, 2.4 mmol) in DCM was added dropwise. The mixture was stirred for 10 min at RT and monitored by TLC (EtOAc:Hex = 3:1). Upon completion of the reaction, the mixture filtered and the solvent was removed under reduced pressure. The residue was dissolved in DMF. Then, Ethanol amine (135 mg, 2.2 mmol) was added. The mixture was stirred for 10 min at RT and monitored by TLC (EtOAc:Hex = 3:1). After completion the reaction was diluted with EtOAc and washed with ammonium chloride saturated solution. The organic layer was dried over magnesium sulfate and the solvent was removed under reduced pressure. The crude product was purified by column chromatography on silica gel (EtOAc:Hex = 3:2) to give compound **4** (570 mg, 84%) in the form of white powder.

RP-HPLC assay, C-18 analytical column, Wavelength; 279 nm, Eluent; acetonitrile:water using isocart elution with 12% acetonitrile, Flow rate; 1 ml/min, R.T; 6.26 min, Purity >95%. ^1^HNMR (400MHz, CD_3_OD) δ = 7.37 (2H, d, J = 9 Hz); 7.19 (1H, dd, J = 9, J = 2 Hz); 7.05-6.99 (2H, m); 6.86 (1H, t, J = 8 Hz); 6.37 (1H, d, J = 8 Hz); 3.67 (2H, s); 3.57 (2H, t, J = 5Hz); 3.29 (2H, t, J = 5 Hz). ^13^C(400MHz, CDCl_3_) δ=173.0, 144.2, 138.4, 133.1, 131.2, 130.3, 128.4, 126.6, 126.2, 121.9, 117.1, 60.8, 42.9, 39.9. HRMS (MALDI-TOF): calculated for C_16_H_16_Cl_2_N_2_O_2_ 361.0481 [M+Na^+^], found 361.0466.

# Compound 5

Compound **4** (200 mg, 0.59 mmol) was dissolved in DCM. Then, nitrated silica gel^2^ (900 mg, 2 eq) was added. The reaction was stirred at RT for 2 hours and monitored by TLC (EtOAc: Hex = 7:3). Upon completion of the reaction, the silica was filtered and the solvent was removed under reduced pressure. The crude product was purified by column chromatography on silica gel (EtOAc: Hex = 7:3) to give compound **5 (**50 mg, 20%) in the form of a yellow powder.

RP-HPLC assay, C-18 analytical column, Wavelength; 368 nm, Eluent; acetonitrile:water using isocart elution with 12% acetonitrile, Flow rate; 1 ml/min, R.T; 10.85 min, Purity >95%. ^1^HNMR (400MHz, CD_3_OD) δ = 8.39 (s, 2H); 8.24 (d, J = 2.5 Hz, 1H); 8.06 (dd, J = 9.0, 2.5 Hz, 1H); 6.71 (d, J = 9.0 Hz, 1H); 3.82 (s, 2H); 3.63 (t, J = 5.0 Hz, 2H); 3.35 (t, J = 5.0 Hz, 2H). ^13^C(400MHz, CDCl_3_) δ=172.2, 149.0, 144.4, 142.9, 142.1, 130.3, 127.4, 127.3, 125.9, 124.7, 118.4, 60.7, 43.0, 40.1. HRMS (ESI-MS): calculated for C_16_H_14_Cl_2_N_4_O_6_ 451.0182 [M+Na^+^], found 451.0191.

# Compound 6

Compound **4** (200 mg, 0.59 mmol) was dissolved in DCM. Then, nitrated silica gel^2^ (1.35 gr, 3 eq) was added. The reaction was stirred at RT for 2 hours and monitored by TLC (EtOAc: Hex = 7:3). Upon completion of the reaction, the silica was filtered and the solvent was removed under reduced pressure. The crude product was purified by column chromatography on silica gel (EtOAc: Hex = 7:3) to give compound **6 (**80 mg, 30%) in the form of a yellow powder.

RP-HPLC assay, C-18 analytical column, Wavelength; 375 nm, Eluent; acetonitrile:water using isocart elution with 12% acetonitrile, Flow rate; 1 ml/min, R.T; 10.16 min, Purity >95%. ^1^HNMR (200MHz, CD_3_OD) δ = 8.59 (1H, d, J = 3 Hz); 8.44 (1H, d, J = 3 Hz); 8.22 (2H , s); 3.86 (2H, s); 3.56 (2H, t, J = 5 Hz); 3.27 (2H, t, J = 5 Hz). ^13^C(400MHz, CDCl_3_) δ=171.8, 143.4, 142.8, 142.2, 141.6, 142.0, 134.4, 130.5, 127.3, 125.8, 121.5, 60.6, 43.1, 40.3. HRMS (ESI-MS): calculated for C_16_H_13_Cl_2_N_5_O_8_ 496.0033 [M+Na^+^], found 496.0053.

# Compound 7

Compound **4** (100 mg, 0.30 mmol) was dissolved in DCM. Then, nitrated silica gel^2^ (250 mg , 1 eq) was added. The reaction was stirred at RT for 2 hours and monitored by TLC (EtOAc: Hex = 7:3). Upon completion of the reaction, the silica was filtered and the solvent was removed under reduced pressure. The crude product was purified by column chromatography on silica gel (EtOAc: Hex = 7:3) to give compound **7 (**30 mg, 25%) in the form of a yellow powder.

RP-HPLC assay, C-18 analytical column, Wavelength; 367 nm, Eluent; acetonitrile:water using isocart elution with 12% acetonitrile, Flow rate; 1 ml/min, R.T; 8.05 min, Purity >95%. ^1^HNMR (200MHz, CD_3_OD) δ = 8.15 (1H, d, J = 3 Hz); 7.94 (1H, dd, J = 9, 3 Hz); 7.49 (2H, d, J = 9 Hz); 7.23 (1H, t, J = 8.5 Hz); 6.30 (1H, d, J = 9.0 Hz); 3.72 (2H, s); 3.59 (2H , t, J = 5 Hz); 3.30 (2H, t, J = 5.0 Hz). ^13^C(400MHz, CDCl_3_) δ=171.5, 151.1, 140.1, 135.9, 133.6, 130.6, 129.5, 127.7, 125.4, 124.4, 114.2, 60.8, 42.9, 40.3. HRMS (MALDI-TOF): calculated for C_16_H_15_Cl_2_N_3_O_4_ 406.0331 [M+Na^+^], found 406.0341.

**(b) General procedure for preparation of diclofenac ester.**

**Compound 8**. The procedure for esterification of diclofenac is demonstrated for compound 8. This compound has been previously described^3-9^. Diclofenac (50 mg, 0.17 mmol) was dissolved in dry DCM. Catalytic amount of DMAP and diethylene glycol (0.08 ml, 0.85mmol) were added. The stirred mixture was cooled to 0ºC, and DCC (52.6 mg, 0.255 mmol) dissolved in DCM was added dropwise. The suspension was then stirred at 0ºC for 30 min and monitored by TLC (EtOAc:He=1:1). The solid was removed by filtration and washed with DCM. The filtrate was concentrated under reduced pressure and chromatographed over silica gel to afford the pure ester compound 8 (43 mg, 66%).

RP-HPLC assay, C-18 analytical column, Wavelength; 275 nm, Eluent; acetonitrile:water using gradient elution 0-20 minutes starting with 10% to 100% acetonitrile, 20-25 minutes 100% acetonitrile, Flow rate; 1 ml/min, R.T; 18.04 min, Purity >95%. ^1^HNMR (200 MHz, CDCl_3_): δ=7.35 ppm (2H, d, J = 8 Hz); 7.19-7.06 (2H, m); 6.99 (1H, d, J = 8 Hz); 6.96 (1H, d, J = 8 Hz.); 6.56 (1H, d, J = 8 Hz); 4.35 (2H, m); 3.8 (2H, s); 3.75-3.65 (4H, m); 3.57-3.53 (2H, m). ^13^C(400MHz, CDCl_3_) δ=173.0, 143.5, 138.5, 131.6, 130.3, 129.6, 128.8, 125.0, 124.8, 122.8, 119.0, 73.1, 69.7, 65.0, 62.5, 39.3. HRMS (MALDI-TOF): calculated for C_18_H_19_Cl_2_NO_4_ 406.0583 [M+Na^+^], found 406.0575.

**Compound 11.** This compound was synthesized as described in procedure (b). Yield 80%. RP-HPLC assay, C-18 analytical column, Wavelength; 275 nm, Eluent; acetonitrile:water using gradient elution 0-20 minutes starting with 10% to 100% acetonitrile, 20-25 minutes 100% acetonitrile, Flow rate; 1 ml/min, R.T; 21.43 min, Purity >95%. ^1^HNMR (200 MHz, CDCl_3_): δ=7.33 ppm (2H, d, J = 8 Hz); 7.22 (1H, dd, J = 7.4, J = 1.6 Hz); 7.12 (1H, dt, J = 7.6, J = 1.6 Hz); 6.98-6.92 (2H, m); 6.55 (1H, d, J = 8 Hz); 3.93 (2H, d, J = 6.8 Hz); 3.82 (2H, s). ^13^C(400MHz, CDCl_3_) δ=173.3, 143.5, 138.7, 131.6, 130.2, 129.6, 128.7, 125.3, 124.7, 122.7, 119.1, 72.1, 39.3, 28.5, 19.8. HRMS (CI): calculated for C_18_H_19_Cl_2_NO_2_ 352.0793 [M+H^+^], found 352.0871.

**Compound 12**. This compound was synthesized as described in procedure (b). Yield 53%. RP-HPLC assay, C-18 analytical column, Wavelength; 275 nm, Eluent; acetonitrile:water using gradient elution 0-20 minutes starting with 10% to 100% acetonitrile, 20-25 minutes 100% acetonitrile, Flow rate; 1 ml/min, R.T; 16.89 min, Purity >95%. ^1^HNMR (200 MHz, CDCl_3_): δ=7.35 ppm (2H, d, J = 8 Hz); 7.19-7.06 (2H, m); 6.99 (1H, d, J=8); 6.96 (1H, d, J=8.); 6.56 (1H, d, J=8); 4.34-4.29 (2H, m); 3.85 (2H, s); 3.75-3.70 (2H, m); 3.61-3.59 (2H, m); 3.58-3.50 (2H, m); 3.37 (3H, s). ^13^C(400MHz, CDCl_3_) δ=173.2, 143.6, 138.6, 131.7, 130.3, 129.6, 128.8, 125.1, 124.8, 122.8, 119.1, 72.6, 71.3, 69.8, 65.2, 59.8, 39.3. HRMS (MALDI-TOF): calculated for C_19_H_21_Cl_2_NO_4_ 420.0739 [M+Na^+^], found 420.0732.

**Compound 13a**

Diclofenac (100 mg, 0.338 mmol) was dissolved in DCM. Then, N-boc-diglycolamine^1^ (70 mg, 0.34 mmol) and DMAP (4 mg, 0.034 mmol) were added. To this mixture DCC (139 mg, 0.68 mmol) DCM was added dropwise. The reaction was stirred at room temperature for 1 hour and monitored by TLC (EtOAc:He = 1:1). Upon completion of the reaction, the mixture was filtered and the solvent was removed under reduced pressure. The crude product was purified by column chromatography on silica gel (EtOAc:He = 1:3) to give compound **13a** (148 mg, 90%) in the form of a color less oil.

^1^HNMR (200 MHz, CDCl_3_): δ = 7.34ppm (2H, d, J = 8 Hz); 7.28 (1H, dd, J = 8, J = 2 Hz); 7.14 (1H, dt, J = 7.4, 1.5 Hz); 7.02-6.93 (2H, m); 6.57 (1H, d, J = 8 Hz,); 4.31-4.26 (2H, m); 3.84 (2H, s) 3.68-3.63 (2H, m); 3.47 (2H , t, J = 5 Hz); 3.27 (2H , q, J = 5 Hz); 1.43 (s, 9H).

**Compound 13**

Compound **13a** (127 mg, 0.26mmol) was dissolved in 10 ml DCM. Then, 3ml of HCl (1M) in ether solution was added. The mixture was stirred at room temperature overnight and monitored by TLC (EtOAc:MeOH = 9:1). Upon completion of the reaction, the solvents were removed under reduced pressure to result in compound **13** (29 mg, 27%) in the form of a white powder which was used without further purification.

RP-HPLC assay, C-18 analytical column, Wavelength; 275 nm, Eluent; acetonitrile:water using gradient elution 0-20 minutes starting with 10% to 100% acetonitrile, 20-25 minutes 100% acetonitrile, Flow rate; 1 ml/min, R.T; 9.19 min, Purity >95%. ^1^HNMR (200 MHz, CDCl_3_): δ = 7.32 ppm (2H, d, J = 8 Hz); 7.26-7.22 (1H, m); 7.12 (1H, t, J = 8 Hz); 7.0-6.92 (2H, m); 6.51 (1H, d, J = 8 Hz); 4.30 (2H, bs); 3.84 (2H, s); 3.76 (4H, bs); 3.25 (2H, bs). ^13^C(400MHz, CDCl_3_) δ=173.38, 143.5, 138.5, 131.8, 130.3, 129.8, 128.8, 125.0, 124.8, 122.8, 118.9, 69.6, 67.7, 65.0, 40.3, 39.2. HRMS (ESI-MS): calculated for C_18_H_21_Cl_2_N_2_O_3_^+^ 383.0924, found 383.0422.

**Compound 14**

Diclofenac (200 mg, 0.67 mmol) was dissolved in DCM. Then, oxiran-2-ylmethanol (60 mg, 0.81 mmol) and DMAP (8 mg, 0.07 mmol) were added. To this mixture DCC (276 mg, 1.34 mmol) in DCM was added dropwise. The reaction was stirred at room temperature overnight and monitored by TLC (EtOAc:He = 1:3). Upon completion of the reaction, the mixture was filtered and the solvent was removed under reduced pressure. The crude product was purified by column chromatography on silica gel (EtOAc:He = 1:3) to give compound **14** (138 mg, 60%) in the form of a white powder.

RF-HPLC assay, C-18 analytical column, Wavelength; 275 nm, Eluent; acetonitrile:water using gradient elution 0-20 minutes starting with 10% to 100% acetonitrile, 20-25 minutes 100% acetonitrile, Flow rate; 1 ml/min, R.T; 14.88 min, Purity >95%. ^1^HNMR (200 MHz, CDCl_3_): δ = 7.34ppm (2H, d, J = 8 Hz); 7.28 (1H, dd, J = 8, J = 2 Hz); 7.14 (1H, dt, J = 7.4, J = 1.5 Hz); 7.02-6.93 (2H, m); 6.57 (1H, d, J = 8 Hz); 4.49 (1H, dd, J = 12, J = 3 Hz); 4.01 (1H, dd, J = 12, J = 6 Hz); 3.88 (2H, s); 3.21 (1H, m); 2.82 (1H, t, J = 6 Hz); 2.62 (1H, dd, J = 4.8, 2.6 Hz). ^13^C(400MHz, CDCl_3_) δ=172.8, 143.5, 138.5, 131.7, 130.2, 129.8, 128.8, 125.0, 124.8, 122.9, 119.2, 66.6, 49.9, 45.4, 39.1**.** HRMS (ESI-MS): calculated for C_17_H_15_Cl_2_NO_3_ 374.0321 [M+Na^+^], found 374.0285.

Compound 9

Diclofenac (300 mg, 1 mmol) was dissolved in DCM. Then, Ethylene glycol (300 mg, 5 mmol) and DMAP (8 mg, 0.07 mmol) were added. To this mixture DCC (240 mg, 1.2 mmol) in DCM was added dropwise. The mixture was stirred for 10 min at RT and monitored by TLC (EtOAc:Hex = 1:1). Upon completion of the reaction, the mixture was filtered and the solvent was removed under reduced pressure. The crude product was purified by column chromatography on silica gel (EtOAc:Hex = 1:3) to give compound **9 (**270 mg, 80%) in the form of white powder.

RP-HPLC assay, C-18 analytical column, Wavelength; 275 nm, Eluent; acetonitrile:water using gradient elution 0-20 minutes starting with 10% to 100% acetonitrile, 20-25 minutes 100% acetonitrile, Flow rate; 1 ml/min, R.T; 16.83 min, Purity >95%. ^1^HNMR (200MHz, CD_3_OD) δ = 7.37 (2H, d, J = 8 Hz); 7.19 (1H, dd, J = 8, J = 2.5 Hz); 7.09-7.00 (2H, m); 6.86 (1H, t, J = 8 Hz); 6.37 (1H, d, J = 8 Hz); 4.18 (2H, t, J = 5 Hz); 3.82 (2H , s); 3.72 (2H, t, J = 5 Hz). ^13^C(400MHz, CDCl_3_) δ=173.4, 143.5, 138.5, 131.6, 130.3, 129.6, 128.9, 125.2, 124.8, 122.9, 119.1, 67.7, 61.9, 39.3. HRMS (ESI-MS): calculated for C_16_H_15_Cl_2_NO_3_ 362.0321 [M+Na^+^], found 362.0313.

# Compound 15

Compound **9** (150 mg, 0.45 mmol) was dissolved in DCM. Then, nitrated silica gel^2^ (375 mg, 1eq) was added. The reaction was stirred at RT for 10 minutes and monitored by TLC (EtOAc: Hex = 1:1). Upon completion of the reaction, the silica was filtered and the solvent was removed under reduced pressure. The crude product was purified by column chromatography on silica gel (EtOAc: Hex = 2:3) to give compound **15** (100 mg, 60%) in the form of an orange powder.

RP-HPLC assay, C-18 analytical column, Wavelength; 365 nm, Eluent; acetonitrile:water using isocart elution with 12% acetonitrile, Flow rate; 1 ml/min, R.T; 7.42 min, Purity >95%. ^1^HNMR (200MHz, CD_3_OD) δ = 8.13 (1H, d, J = 2 Hz); 7.93 (1H, dd, J = 9, J = 2 Hz); 7.49 (2H, d, J = 8.5 Hz); 7.27 (1H, t, J = 8.5 Hz); 6.23 (1H, d, J = 9.0 Hz); 4.21 (2H, t, J = 5 Hz); 3.89 (2H, s); 3.74 (2H, t, J = 5 Hz). ^13^C(400MHz, CDCl_3_) δ=172.6, 149.8, 141.7, 136.0, 132.5, 129.9, 128.0, 127.6, 122.5, 120.0, 116.2, 68.1, 61.6, 39.4. HRMS (ESI-MS): calculated for C_16_H_14_Cl_2_N_2_O_3_ 407.0171 [M+Na^+^], found 407.0132.

**(c) General procedure for esters 19 and 20**

Isobutyl alcohol (1.5 mmol) and DCC (1.0 mmol) were added to the corresponding acid (1.0 mmol) dissolved in 10 ml of dichloromethane. The mixture was stirred for 1 hour and monitored by TLC (EtOAc:He=1:3). After completion the reaction was diluted with EtOAc and washed with ammonium chloride saturated solution. The organic layer was dried over magnesium sulfate and the solvent was removed under reduced pressure. The crude product was purified by column chromatography on silica gel to give the compounds **19** and **20**.

**Compound 19.** This compound was synthesized as described in procedure (c). Yield 83%. RP-HPLC assay, C-18 analytical column, Wavelength; 275 nm, Eluent; acetonitrile:water using gradient elution 0-20 minutes starting with 10% to 100% acetonitrile, 20-25 minutes 100% acetonitrile, Flow rate; 1 ml/min, R.T; 18.53 min, Purity >95%. ^1^HNMR (200 MHz, CDCl_3_): δ = 7.51 (1H, dd, J = 2, J = 8 Hz); 7.28-6.98 (5H, m); 6.71 (1H, dt, J = 2, J = 8 Hz); 2.05 (1H, m); 0.97 (6H, d, J = 6.8 Hz).. ^13^C(400MHz, CDCl_3_) δ=169.4, 149.2, 141.7, 136.3, 134.8, 132.3, 127.6, 126.3, 123.4, 121.1, 117.7, 114.6, 112.3, 71.5, 28.7, 20.0, 15.8. HRMS (CI): calculated for C_18_H_20_ClNO_2_ 318.1183 [M+H^+^], found 318.1260.

**Compound 20.** This compound was synthesized as described in procedure (c). Yield 75%. RP-HPLC assay, C-18 analytical column, Wavelength; 275 nm, Eluent; acetonitrile:water using gradient elution 0-20 minutes starting with 10% to 100% acetonitrile, 20-25 minutes 100% acetonitrile, Flow rate; 1 ml/min, R.T; 17.30 min, Purity >95%. ^1^HNMR (200 MHz, CDCl_3_): δ = 7.60 (1H, dd, J = 2, J = 8 Hz); 7.49 (2H, d, J = 8 Hz); 7.43-7.26 (3H, m); 7.18 (1H, bs); 6.93 (1H, dt, J = 2, J = 8); 2.03 (1H, m); 0.95 (6H, d, J = 7.0 Hz). ^13^C(400MHz, CDCl_3_) δ=169.2, 147.5, 142.5, 134.9, 132.7, 132.4, 130.6, 125.2, 123.8, 120.2, 119.9, 118.3, 115.1, 114.1, 71.7, 28.6, 20.0. HRMS (CI): calculated for C_18_H_18_F_3_NO_2_ 338.1290 [M+H^+^], found 338.1367.

**References**

1. Kim YS, Kim KM, Song R, Jun MJ, Sohn YS J. (2001) *Inorg. Biochem 87: 157-163.*

2. Fleming SA, Ridges MD, Jensen AW J. (1996) *Label. Compd. Radiopharm. 38: 13-18.*

3. Orjales VA, Mosquera Pestana R. (1986) *CODEN: SPXXAD ES 548226 A1 19860316 Patent written in Spanish. Application: ES 85-548226 19851025.*

4. Metz G, Raeuchle K, Erdmann M. (1989) *CODEN: GWXXAW DE 3811118 C1 19891012 Patent written in German. Application: DE 88-3811118 19880331.*

5. Bonina FP, Puglia C, Barbuzzi T, de Caprariis P, Palagiano F, Rimoli MG, Saija A. (2001) *Eur. J. Pharma.Sci.14: 123-134.*

6. Bonina F, Puglia C, Santagati NA, Saija A, Tomaino A, Tita B. (2002) *Pharmazie 57: 552-555.*

7. Andersson J, Belli A, Cannata V, Hedberg M, Palmgren A, Schuldei S, Stroem M, Villa M (2004) *CODEN: PIXXD2 WO 2004026808 A1 20040401. Patent written in English. Application: WO 2003-SE1465 20030918.*

8. Attali B, Peretz A. (2004) *CODEN: PIXXD2 WO 2004035037 A2 20040429. Patent written in English. Application: WO 2003-IL855 20031021.*

9. Attali B, Peretz A. (2005) *CODEN: USXXCO US 2005250833 A1 20051110 Patent written in English. Application: US 2005-110669 20050421.*
